# Supplementary material for: Static Culture Combined with Aeration in Biosynthesis of Bacterial Cellulose
Source: Polymers (Basel). 2021 Dec 3;13(23):4241. doi: 10.3390/polym13234241 (PMC8659626; doi:10.3390/polym13234241)
Supplement: Supplementary file 1 [file polymers-13-04241-s001.zip › polymers-1487060-supplementary.pdf]

Supplementary Materials

Static Culture Combined with Aeration in Biosynthesis of Bacterial Cellulose

Nadezhda A. Shavyrkina [1,2], Ekaterina A. Skiba [1,2], Anastasia E. Kazantseva [1], Evgenia K. Gladysheva [1], Vera V. Budaeva [1,*], Nikolay V. Bychin [1], Yulia A. Gismatulina [1], Ekaterina I. Kashcheyeva [1], Galina F. Mironova [1], Anna A. Korchagina [1], Igor N. Pavlov [1,2] and Gennady V. Sakovich [1]

1 Bioconversion Laboratory, Institute for Problems of Chemical and Energetic Technologies, Siberian Branch of the Russian Academy of Sciences (IPCET SB RAS), Biysk 659322, Altai Krai, Russia; 32nadina@mail.ru (N.A.S.); eas08988@mail.ru (E.A.S.); sitnikova97.97@mail.ru (A.E.K.); evg-gladysheva@yandex.ru (E.K.G.); nbych@yandex.ru (N.V.B.); julia.gismatulina@mail.ru (Y.A.G.); makarova@ipcet.ru (E.I.K.); yur_galina@mail.ru (G.F.M.); yakusheva89_21.ru@mail.ru (A.A.K.); pawlow-in@mail.ru (I.N.P.); admin@ipcet.ru (G.V.S.)

2 Biysk Technological Institute, Polzunov Altai State Technical University, Biysk 659305, Altai Krai, Russia

* Correspondence: budaeva@ipcet.ru

Table of Contents

Figure S1. Photographs of BC samples

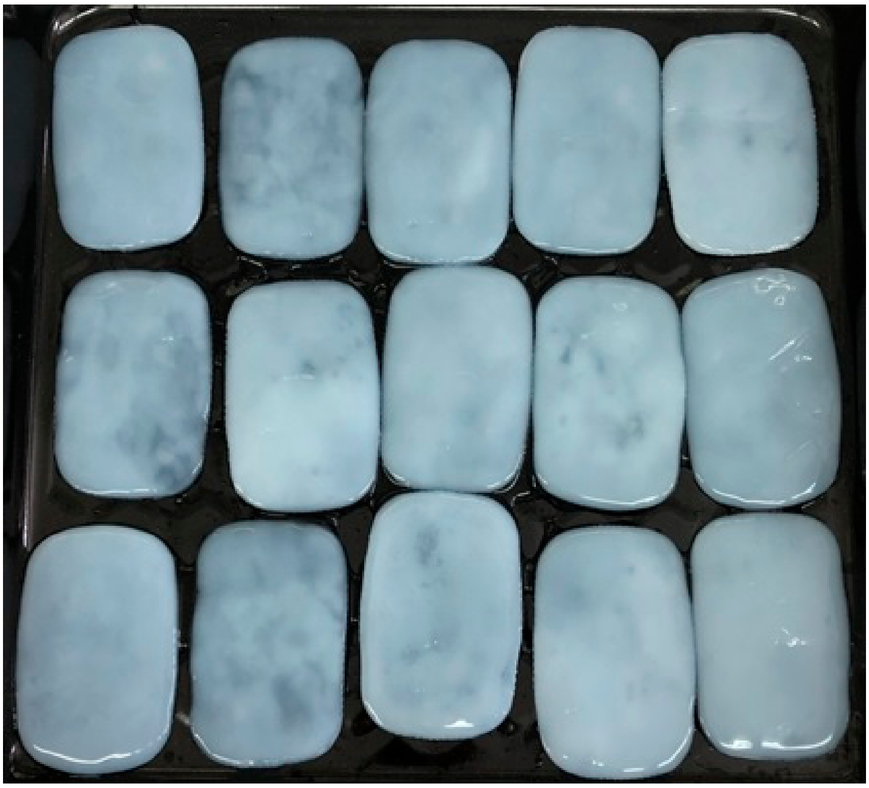

(A)

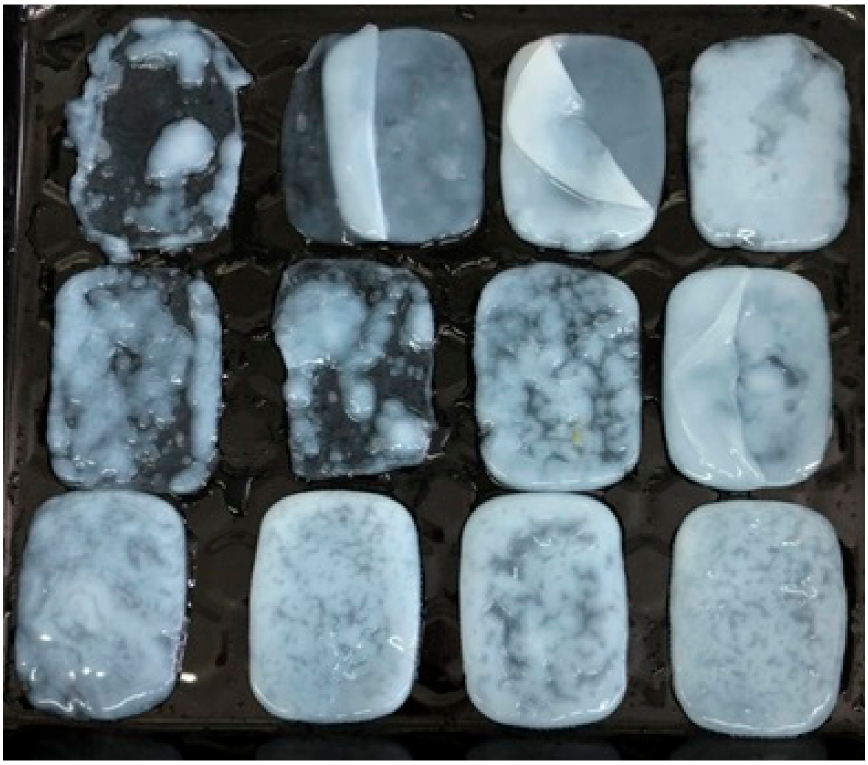

(B)

Figure S1. Photographs of BC samples obtained with: (**A**) no aeration or far away from the air streams and (**B**) at an air flow rate of 6.3 L/min, nearby the air streams.
